# Atingimento das Metas de Colesterol LDL em Pacientes com Histórico de Infarto Agudo do Miocárdio: Estudo Transversal do Mundo Real

**DOI:** 10.36660/abc.20230242

**Published:** 2024-02-22

**Authors:** Daniel A. Gomes, Mariana Sousa Paiva, Pedro Freitas, Francisco Albuquerque, Maria Rita Lima, Rita Reis Santos, João Presume, Marisa Trabulo, Carlos Aguiar, Jorge Ferreira, António M. Ferreira, Miguel Mendes

**Affiliations:** 1 Hospital de Santa Cruz Centro Hospitalar de Lisboa Ocidental Lisboa Portugal Hospital de Santa Cruz – Centro Hospitalar de Lisboa Ocidental, Lisboa – Portugal; 2 Hospital da Luz Lisboa Portugal Hospital da Luz, Lisboa – Portugal

**Keywords:** Colesterol LDL, Aterosclerose, Prevenção Secundária

## Abstract

**Fundamento:**

As diretrizes da Sociedade Europeia de Cardiologia recomendam um nível de colesterol LDL (LDL-C) < 55 mg/dL para pacientes com doença cardiovascular estabelecida. Embora a fórmula de Friedewald ainda seja amplamente utilizada para estimar o LDL-C, a fórmula mais recente de Martin-Hopkins mostrou maior precisão.

**Objetivos:**

Nosso objetivo foi avaliar: A) a proporção de pacientes que atingiram a meta de LDL-C e as terapias utilizadas em um centro terciário; B) o impacto da utilização do método de Martin-Hopkins em vez do método de Friedewald na proporção de pacientes controlados.

**Métodos:**

Estudo transversal monocêntrico, incluindo pacientes consecutivos pós-infarto do miocárdio, acompanhados por 20 cardiologistas, em um hospital terciário. Os dados foram coletados retrospectivamente de consultas clínicas realizadas após abril de 2022. Para cada paciente, os níveis de LDL-C e o atingimento das metas foram estimados a partir de um perfil lipídico ambulatorial, utilizando as fórmulas de Friedewald e Martin-Hopkins. Um valor-p bicaudal < 0,05 foi considerado estatisticamente significativo para todos os testes.

**Resultados:**

Foram incluídos 400 pacientes (com 67 ± 13 anos, 77% do sexo masculino). Utilizando a fórmula de Friedewald, a mediana de LDL-C sob terapia foi de 64 (50-81) mg/dL, e 31% tinham LDL-C dentro da meta. Estatinas de alta intensidade foram usadas em 64% dos pacientes, 37% estavam em uso de ezetimiba e 0,5% estavam em uso de inibidores de PCSK9. A terapia combinada de estatina de alta intensidade + ezetimiba foi utilizada em 102 pacientes (26%). A aplicação do método de Martin-Hopkins reclassificaria um total de 31 pacientes (7,8%). Entre aqueles considerados controlados pela fórmula de Friedewald, 27 (21,6%) teriam LDL-C estimado por Martin-Hopkins acima da meta.

**Conclusões:**

Menos de um terço dos pacientes pós-infarto do miocárdio apresentaram LDL-C dentro da meta. A aplicação da fórmula de Martin-Hopkins reclassificaria um quinto dos pacientes presumivelmente controlados no grupo de pacientes não controlados.

**Figure:**
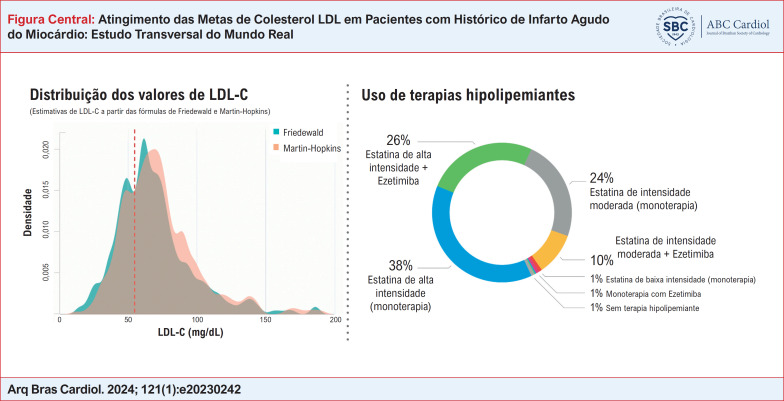
A: Gráfico de densidade de LDL-C estimada pelas fórmulas de Friedewald e Martin-Hopkins. No geral, 31% e 26% dos pacientes apresentaram LDL-C dentro da meta, respectivamente. B: Gráfico circular exibindo a prescrição de terapias hipolipemiantes. Menos de dois terços dos pacientes (64%) receberam prescrição de estatinas de alta intensidade. A intolerância às estatinas foi relatada em cinco casos.

## Introdução

O colesterol de lipoproteína de baixa densidade (LDL-C) é um fator de risco bem estabelecido e modificável para doença cardiovascular aterosclerótica (DCVA).^1-3^ Atualmente, as diretrizes da Sociedade Europeia de Cardiologia (ESC)/Sociedade Europeia de Aterosclerose (EAS) de 2019 para o tratamento de dislipidemias recomendam uma redução ≥ 50% do nível basal e uma meta de LDL-C < 55 mg/dL para prevenção secundária (recomendação classe IA).^4^ O colesterol não HDL (não HDL-C) é considerado uma meta secundária, com meta de tratamento < 85 mg/dL em pacientes com risco muito alto de eventos cardiovasculares.^4^ A terapia hipolipemiante intensiva é, portanto, a chave para reduzir o risco de futuros eventos cardiovasculares.^4^ Ensaios anteriores forneceram evidências de que as terapias hipolipemiantes de alta intensidade são seguras e mais eficazes do que os medicamentos de baixa intensidade na redução da mortalidade por todas as causas e de eventos cardiovasculares recorrentes em pacientes com DCVA.^5^ As recomendações internacionais defendem o uso de estatinas de alta intensidade (um substituto para alcançar uma redução de ≥ 50% do LDL-C) como farmacoterapia de primeira linha para reduzir o LDL-C e o risco cardiovascular.^4^ Em pacientes com risco muito alto de eventos cardiovasculares, incluindo aqueles com DCVA estabelecida, que não atingem as metas estabelecidas com a dose máxima tolerada de uma estatina, recomenda-se a associação de ezetimiba e, se necessário, de um inibidor de PCSK9.^4^ Apesar de estar cada vez mais reconhecido que os maiores benefícios absolutos das terapias hipolipemiantes ocorrem em indivíduos com maior risco, como aqueles com histórico de DCVA, ainda existe um contraste significativo entre as recomendações das diretrizes e a prática clínica do mundo real.^6,7^

Embora o LDL-C plasmático possa ser medido diretamente, na prática clínica, ele é mais frequentemente calculado a partir de um perfil lipídico padrão, desde que o colesterol total (CT) esteja distribuído principalmente entre LDL-C, lipoproteína de alta densidade (HDL-C) e lipoproteína de densidade muito baixa (VLDL-C).^4^ A fórmula de Friedewald é o método mais utilizado para estimar o LDL-C.^8^ Usando este método, o LDL-C é calculado subtraindo-se tanto o HDL-C quanto os triglicerídeos (TG) /5 (como uma estimativa para o VLDL-C) do CT.^8^ Embora conveniente, esta fórmula apresenta diversas limitações. Como um fator fixo de 5 é usado para estimar o VLDL-C, a fórmula de Friedewald é propensa a maior imprecisão em pacientes com níveis baixos de LDL-C e/ou níveis elevados de TG, resultando em uma subestimação significativa do LDL-C.^9^ Além disso, a fórmula de Friedewald deve ser utilizada com cautela em pacientes com risco alto ou muito alto de DCVA devido a sua subestimação significativa do LDL-C, pois pode impedir os médicos de intensificar a terapia hipolipemiante recomendada pelas diretrizes. A fórmula de Martin-Hopkins é um método mais recente para estimar o LDL-C e tem mostrado maior precisão do que a de Friedewald, especialmente em LDL-C baixo e TG alto.^10^ Ao contrário da fórmula de Friedewald, a fórmula de Martin-Hopkins divide o TG por um fator ajustável com base nos níveis de não HDL-C e TG do paciente. Embora a fórmula de Friedewald ainda seja a mais utilizada, as Diretrizes para Colesterol da American Heart Association/American College of Cardiology de 2018 forneceram uma recomendação de classe IIa (Nível de evidência C) para o uso da fórmula de Martin-Hopkins ou medição direta em pacientes com LDL-C < 70 mg/dL.^11^ É importante ressaltar que ambas as fórmulas foram validadas apenas para pacientes com TG < 400 mg/dL.^8,10,12^ Em níveis mais elevados de TG, os quilomícrons se acumulam e podem alterar a relação entre TG e VLDL-C.^11^ Nestas circunstâncias, deve ser utilizada a medição direta do LDL-C.

## Objetivos

Os objetivos deste estudo foram: A) avaliar a proporção de pacientes que atingiram sua meta de LDL-C e as terapias utilizadas em um hospital terciário, e B) avaliar o impacto do uso do método de Martin-Hopkins em vez da fórmula de Friedewald sobre a proporção de pacientes controlados.

## Métodos

### População do estudo

Este foi um estudo transversal, monocêntrico, incluindo pacientes consecutivos pós-infarto do miocárdio, acompanhados por 20 cardiologistas diferentes (cada um acompanhando 20 pacientes), em um hospital terciário, que tiveram consulta clínica após abril de 2022. Os pacientes foram considerados elegíveis quando cumpriram todos os seguintes critérios: A) infarto agudo do miocárdio tipo 1 ≥ 6 meses antes da consulta; B) perfil lipídico em jejum, ambulatorial, disponível, realizado no laboratório do hospital; C) terapia hipolipemiante estável por ≥ 6 semanas antes da análise de sangue; D) nível medido de TG em jejum < 400 mg/dL.

### Dados demográficos, clínicos e laboratoriais

Dados demográficos, clínicos e laboratoriais, bem como medicamentos, foram coletados retrospectivamente dos prontuários eletrônicos de consultas clínicas dos pacientes. Também foram coletados dados referentes às mudanças terapêuticas e agendamento de acompanhamento. Fatores de risco cardiovascular e infarto do miocárdio foram definidos de acordo com recomendações atuais.^13,14^ As medições laboratoriais de CT, HDL-C e TG foram realizadas a partir de amostras de sangue em jejum dos pacientes, no laboratório central do hospital.

Este estudo foi conduzido de acordo com emenda da Declaração de Helsinque. Os dados clínicos foram coletados pelo cardiologista responsável pelo paciente e anonimizados de forma irreversível antes de sua introdução na base de dados à disposição dos investigadores. O consentimento informado dos pacientes foi dispensado no âmbito do Programa de Certificação de Qualidade da Autoridade Nacional de Saúde para o desenvolvimento de uma Auditoria Interna. Não houve ausência de pacientes ou dados. Os dados foram relatados de acordo com as diretrizes de relatórios RECORD.^15^

### Terapias hipolipemiantes e metas de LDL-C

A estatina de baixa intensidade foi definida como uma dose diária de sinvastatina 10 mg, pravastatina 10-20 mg, lovastatina 20 mg ou fluvastatina 20-40 mg. A estatina de intensidade moderada foi definida como uma dose diária de sinvastatina 20-40 mg, atorvastatina 10-20 mg, rosuvastatina 5-10 mg, pravastatina 40-80 mg, pitavastatina 1-4 mg, lovastatina 40 mg ou fluvastatina 80 mg. A estatina de alta intensidade foi definida como uma dose diária de rosuvastatina 20-40 mg ou atorvastatina 40-80 mg.^11^ Os pacientes foram considerados intolerantes às estatinas quando claramente indicado nos registros clínicos.

Para cada paciente, os níveis de LDL-C e o de atingimento das metas foram estimados pelas fórmulas de Friedewald e Martin-Hopkins. De acordo com as diretrizes da ESC, os pacientes foram considerados controlados quando em um nível de LDL-C em jejum dentro da meta recomendada (< 55 mg/dL),^4^ ao passo que um nível de colesterol não-HDL < 85 mg/dL foi uma meta secundária.

### Análise estatística

As variáveis categóricas foram relatadas como números e porcentagens. As variáveis contínuas foram descritas como médias e desvio padrão para variáveis com distribuição normal, e medianas e intervalos interquartis para variáveis com distribuição não normal. A normalidade dos dados foi avaliada pelo teste de Kolmogorov-Smirnov. As características clínicas dos subgrupos de interesse foram comparadas por meio do teste χ2 e teste exato de Fisher (quando aplicável) para variáveis dicotômicas; e teste t de Student não pareado ou teste U de Mann-Whitney (quando aplicável) para variáveis contínuas. Modelos de regressão logística univariada e multivariada foram utilizados para explorar fatores associados à não prescrição de estatinas de alta intensidade e ao não atingimento das metas de LDL-C. Apenas variáveis com valor-p < 0,05 foram incluídas no modelo multivariado. Um valor-p bicaudal < 0,05 foi considerado estatisticamente significativo para todos os testes. Todas as análises foram realizadas utilizando o software IBM® SPSS® Statistics (versão 26.0).

## Resultados

### Características dos pacientes e medicação

Um total de 400 pacientes pós-infarto do miocárdio foram incluídos no estudo. A população era composta de 20 grupos de 20 pacientes consecutivos, sendo cada coorte acompanhada por um cardiologista diferente. No geral, o último infarto do miocárdio havia ocorrido dentro de uma mediana de cinco anos (IQR 2-12) antes da consulta, e a experiência em prática clínica mediana dos médicos (ou seja, anos desde a graduação da faculdade de medicina), naquela época, era de sete anos (IQR 5-26). As características demográficas e clínicas dos pacientes estão detalhadas na Tabela 1 e na Tabela Suplementar 1.

**Tabela 1:** Características demográficas, clínicas e laboratoriais da população estudada

		Todos (N = 400)	Nenhuma terapia com estatinas de alta intensidade (N = 144)	Terapia com estatinas de alta intensidade (N = 256)	valor-p
**Anos de idade**		67±13	72±12	65±13	< 0,001
**Sexo masculino**		307 (76,8%)	113 (78,5%)	194 (75,8%)	0,541
**Hipertensão arterial**		266 (66,5%)	100 (69,4%)	166 (64,8%)	0,349
**Diabetes tipo 2**		125 (31,3%)	50 (34,7%)	75 (29,3%)	0,261
**Histórico de tabagismo**		228 (57,0%)	74 (51,4%)	154 (60,1%)	0,089
	Fumante ativo	70 (17,5%)	20 (13,9%)	50 (19,5%)	0,154
**Doença renal crônica** *		72 (18,0%)	32 (22,2%)	40 (15,6%)	0,099
**Anos desde o último IM**		5 (2-12)	10 (5-16)	3 (1-9)	< 0,001
**DCVA em outros territórios**					
	AVC prévio	18 (4,5%)	8 (5,6%)	10 (3,9%)	0,445
	Doença arterial periférica	49 (12,3%)	18 (12,5%)	31 (12,1%)	0,909
**Eventos CV recorrentes**		93 (23,3%)	38 (26,4%)	55 (21,5%)	0,265
**Perfil lipídico sérico**					
	Colesterol total, mg/dL	135 (118-155)	145 (129-166)	130 (114-148)	< 0,001
	HDL-C, mg/dL	45 (38-53)	46 (38-55)	44 (37-52)	0,106
	LDL-C, mg/dL (fórmula de Friedewald)	64 (50-81)	72 (57-92)	62 (47-75)	< 0,001
	Colesterol não-HDL, mg/dL	89 (72-110)	97 (79-118)	86 (69-103)	< 0,001
	TG, mg/dL	107 (82-153)	107 (84-157)	107 (80-153)	0,776

Os valores são apresentados como média ± desvio padrão, mediana (intervalo interquartil) ou *n* (%).

* Doença renal crônica definida como taxa de filtração glomerular estimada < 60 mL/min/1,73 m^2^ (Equação CKD-EPI). DCVA: doença cardiovascular aterosclerótica; CV: cardiovascular; HDL-C: colesterol lipoproteico de alta densidade; LDL-C: colesterol lipoproteico de baixa densidade; IM: infarto do miocárdio; TG: triglicerídeos.

Utilizando a fórmula de Friedewald, a mediana estimada de LDL-C sob terapia foi de 64 mg/dL (IQR 50-81), enquanto foi de 69 mg/dL (IQR 54-86) quando calculada pela fórmula de Martin-Hopkins. Dependendo do método de estimativa utilizado (Martin-Hopkins ou Friedewald), 102 ou 125 pacientes (26% ou 31%, respectivamente) apresentaram LDL-C dentro da meta [ou seja, < 55 mg/dL] (Figura Central). No total, 88% (n = 110) dos pacientes considerados controlados pela fórmula de Friedewald e todos aqueles com LDL-C dentro da meta estimada por Martin-Hopkins atingiram a meta secundária de colesterol não-HDL.

Estatinas de alta intensidade foram utilizadas em 64% dos pacientes e 26% receberam prescrição de estatinas de alta intensidade em associação com ezetimiba (Tabela 2 e Figura Central). A intolerância às estatinas foi relatada em 3,5% (n = 5) dos pacientes que não tomaram doses de alta intensidade. O grupo de pacientes em uso de estatinas de alta intensidade (com ou sem ezetimiba) atingiu níveis plasmáticos mais baixos de LDL-C, calculados pela fórmula de Friedewald (62 [IQR 47-75] vs. 72 [57-92] mg/dL, p< 0,001) e colesterol não-HDL (86 [IQR 69-103] vs. 97 [IQR 79-118] mg/dL, p < 0,001) (Tabela 1). Ao considerar pacientes sob uso de estatina de alta intensidade mais ezetimiba (n = 102), 35% (n = 36) apresentaram LDL-C dentro da meta, enquanto 11% (n = 11) permaneceram acima de 100 mg/dL.

**Tabela 2:** Terapias hipolipemiantes

Terapia hipolipemiante		Toda a coorte (N=400)	LDL-C dentro da meta (N=125)*	LDL-C fora da meta (N=275)*	Valor-p
**Estatinas em monoterapia**		252 (63,0%)	73 (58,4%)	179 (65,1%)	0,199
	Baixa intensidade	3 (0,8%)	0 (0,0%)	3 (1,1%)	0,241
	Intensidade moderada	95 (23,8%)	20 (16,0%)	75 (27,3%)	0,014
	Alta intensidade	154 (38,5%)	56 (44,8%)	98 (35,6%)	0,081
**Ezetimiba em monoterapia**		3 (0,8%)	1 (0,8%)	2 (0,7%)	0,938
**Estatina + ezetimiba**		142 (35,5%)	48 (38,4%)	94 (34,2%)	0,414
	Baixa intensidade	0 (0,0%)	0 (0,0%)	0 (0,0%)	-
	Intensidade moderada	40 (10,0%)	12 (9,6%)	26 (10,2%)	0,857
	Alta intensidade	102 (25,5%)	36 (28,8%)	66 (24,0%)	0,773
**Fibratos**		16 (4,0%)	3 (2,4%)	13 (4,7%)	0,271
**Inibidores de PCSK9**		2 (0,5%)	2 (1,6%)	0 (0,0%)	0,035
**Sem terapia hipolipemiante**		3 (0,8%)	0 (0,0%)	3 (1,1%)	0,241

* LDL-C estimado pela fórmula de Friedewald.

No modelo de regressão logística multivariada, a idade mais avançada dos pacientes e o tempo de atuação do cardiologista foram preditores de não prescrição de estatinas de alta intensidade (Tabela 3). Os fatores associados ao atingimento das metas de LDL-C estão representados na Tabela Suplementar 2.

**Tabela 3:** Análises univariadas e multivariadas explorando fatores associados à não prescrição de estatinas de alta intensidade

	Análise Univariada			Análise Multivariada		
	OR	IC 95%	Valor-p	OR ajustada	IC 95%	Valor-p
Idade, por ano	1,045	1,027-1,064	<0,001	1,041	1,021–1,060	< 0,001
Sexo masculino	0,858	0,526-1,401	0,541			
Hipertensão	0,812	0,524-1,258	0,350			
Diabetes mellitus tipo 2	0,779	0,504-1,205	0,262			
Fumante ativo	0,665	0,378-1,168	0,156			
Anos desde o último IM	0,999	0,996-1,001	0,382			
Doença renal crônica	0,648	0,386-1,088	0,101			
Doença arterial periférica	0,964	0,519-1,792	0,909			
Doença cerebrovascular	0,691	0,266-1,792	0,447			
Eventos CV recorrentes	0,763	0,474-1,229	0,266			
Experiência clínica de cardiologistas: > 10 anos vs. ≤ 10 anos	1,937	1,279-2,932	0,002	1,885	1,230–2,890	0,004

IC: intervalo de confiança; CV: cardiovascular; IM: infarto do miocárdio; OR: razão de chances (odds ratio).

### Método de estimativa de LDL-C

**Figura 1:**
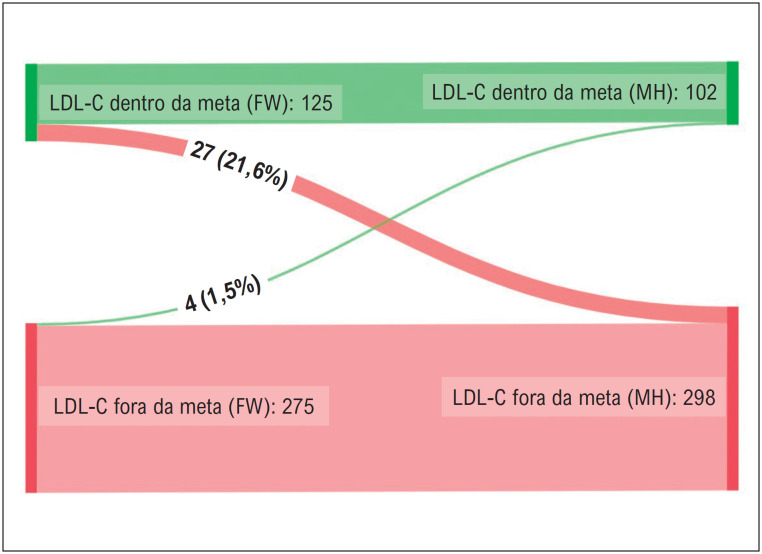
Diagrama de Sankey mostrando a reclassificação dos pacientes, de acordo com o atingimento da meta de LDL-C ao aplicar a fórmula de Martin-Hopkins em vez da de Friedewald. No geral, 22% dos pacientes considerados controlados pela fórmula de Friedewald teriam um LDL-C de Martin-Hopkins acima da meta, enquanto 2% seriam recategorizados como tendo LDL-C dentro da meta.

Aplicando-se a fórmula de Martin-Hopkins em vez da de Friedewald, um total de 31 pacientes (7,8%) seriam reclassificados quanto ao atingimento das metas de LDL-C. Enquanto 27 (21,6%) pacientes considerados controlados pela fórmula de Friedewald teriam LDL-C calculado por Martin-Hopkins acima de 55 mg/dL, 4 (1,5%) pacientes previamente classificados como não controlados teriam LDL-C recalculado dentro da meta (Figura 1). Os pacientes reclassificados apresentaram valores mais elevados de TG (183 [IQR 127-287] vs. 104 [IQR 79-148] mg/dL, p < 0,001) e níveis mais baixos de LDL-C estimado pela fórmula de Friedewald (51 [IQR 46-54] vs. 67 [IQR 61-82] mg/dL, p < 0,001).

### Mudanças de medicação

O ajuste das terapias hipolipemiantes foi feito em 70 (25,5%) dos 275 pacientes com níveis de LDL-C acima da meta. As alterações medicamentosas mais frequentes foram titulação da intensidade ou dose das estatinas (36, 13,1%) e/ou associação de ezetimiba (48, 17,5%). Sete pacientes (2,5%) foram encaminhados para terapia com inibidor de PCSK9. A consulta seguinte de acompanhamento foi agendada dentro de uma mediana de 8 meses (IQR 6-11).

## Discussão

Nesta análise transversal do mundo real, relatamos o padrão de prescrição de terapias hipolipemiantes e o atingimento das metas de LDL-C em pacientes com histórico de infarto do miocárdio, acompanhados em uma clínica de cardiologia de um hospital terciário. Os principais resultados podem ser resumidos da seguinte forma: (1) Houve uma subutilização significativa de terapias hipolipemiantes prontamente disponíveis, com estatinas de alta intensidade sendo prescritas em menos de dois terços dos pacientes; (2) Apenas um em cada três pacientes atingiu a meta de LDL-C; (3) A aplicação da fórmula de Martin-Hopkins em vez da de Friedewald reclassificaria mais de 20% dos pacientes presumivelmente controlados no grupo não controlado.

### Padrão de prescrição e atingimento das metas de LDL-C

O LDL-C é um importante determinante do risco cardiovascular e tem sido, há muito tempo, uma meta primária de tratamento nas recomendações clínicas.^4^ Na verdade, cada redução absoluta de 38,7 mg/dL no LDL-C atingido com estatinas reduz os eventos vasculares maiores em 22% e a mortalidade por todas as causas em 10%.^16^ As diretrizes atuais defendem o uso de terapia hipolipemiante (nomeadamente estatinas de alta intensidade), se não for contraindicada, em todos os pacientes com DCVA estabelecida, independentemente do nível basal de LDL-C, para reduzir a morbidade e a mortalidade, visando uma meta de LDL-C < 55 mg/dL e uma redução de pelo menos 50%.^4^ É importante reconhecer que estas metas são difíceis de obter com estatinas em monoterapia, e que a terapia combinada com ezetimiba é muitas vezes necessária.^4^ Caso os níveis de LDL-C permaneçam acima da meta, recomenda-se a associação com um inibidor de PCSK9.^4^

Neste estudo contemporâneo de pacientes em prevenção secundária de DCVA, 64% receberam estatinas de alta intensidade e apenas 31% apresentaram LDL-C controlado, mesmo que a intolerância às estatinas tenha sido relatada em pouco mais de 1%. Além disso, apesar de ter descoberto que a maioria dos pacientes não apresentava LDL-C dentro da meta, não mais do que um quarto estava sob uso de estatina de alta intensidade mais ezetimiba, e apenas dois dos 36 (6%) pacientes elegíveis, de acordo com as diretrizes ESC/EAS, estavam em uso de um inibidor de PCSK9. Estes resultados ressaltam a grande lacuna entre as diretrizes sociais e a prática clínica no mundo real e, embora desanimadores, estão alinhados a outros estudos observacionais anteriores: (i) a pesquisa EUROASPIRE V relatou que apenas 29% dos pacientes com doença arterial coronariana estabelecida, acompanhados em centros europeus, apresentavam um LDL-C < 70 mg/dL (anteriormente recomendado pelas diretrizes ESC/EAS de 2016);^17^ (ii) O estudo DA VINCI, incluindo pacientes em prevenção primária e secundária, mostrou que um terço atingiu as metas de LDL-C recomendadas pela ESC/EAS em 2019;^18^ (iii) na análise inicial do estudo SANTORINI, apenas 21% dos pacientes com DCVA estabelecida, acompanhados em centros de cuidados primários ou secundários em toda a Europa, apresentavam LDL-C < 55 mg/dL;^19^ (iv) no estudo LATINO, incluindo pacientes acompanhados em centros de cuidados primários e secundários portugueses ao longo de 20 anos, apenas 10% dos pacientes de risco muito alto atingiram a meta de LDL-C da ESC/EAS de 2016.^20^ Ao contrário do que é preconizado nas diretrizes, as estatinas de alta intensidade também foram significativamente subutilizadas nestes estudos de grande escala, variando a sua utilização entre 12% no LATINO e 50% na pesquisa EUROASPIRE V, em pacientes com risco muito alto de eventos cardiovasculares.^17,20^

Notavelmente, os pacientes mais velhos, bem como aqueles que foram acompanhados durante mais tempo, tiveram menos probabilidade de receber terapias de maior intensidade, enquanto, ao contrário do EUROASPIRE V, não foram registadas diferenças relacionadas ao sexo nos padrões de prescrição ou no atingimento das metas de LDL-C. Além disso, os anos de atuação como cardiologista foram um dos preditores de não prescrição de estatinas de alta intensidade. É possível que os médicos mais experientes sejam menos propensos a aderir às novas diretrizes sociais e mais propensos a confiar em evidências clínicas não atualizadas, como cogitado anteriormente.^21,22^

Apenas cerca de 25% dos pacientes não controlados tiveram sua medicação hipolipemiante titulada, e a próxima consulta de acompanhamento foi agendada dentro de um tempo médio de 8 meses, apesar das diretrizes recomendarem a reavaliação do LDL-C dentro de 4-6 semanas e, se necessário, intensificar a terapia.^4^ Esses achados destacam o conceito de inércia terapêutica, que é definida como "falha em avançar ou intensificar a terapia quando as metas terapêuticas não são alcançadas".^23^ Os impulsionadores da inércia terapêutica podem ser divididos em três categorias: relacionados ao provedor (restrições de tempo, demandas concorrentes e falta de conhecimento), relacionadas ao paciente (multimorbidade, preocupações com efeitos colaterais, incompreensão dos regimes de tratamento) e relacionadas ao sistema (questões e custos de saúde).^23,24^ A inércia terapêutica pode, em última análise, aumentar o risco de complicações evitáveis relacionadas à doença e, portanto, todos os esforços devem ser empregados para a reduzi-la.^24^

### Método de estimativa de LDL-C

As diretrizes da ESC/EAS de 2019 não fornecem orientação sobre o método ideal de avaliação de LDL-C (medição direta vs. calculada).^4^ A sua avaliação precisa é, no entanto, essencial, uma vez que as decisões de tratamento são muitas vezes baseadas no atingimento de uma meta específica. Apesar dos métodos diretos estarem cada vez mais disponíveis, a fórmula de Friedewald de 1972 ainda é a mais utilizada para estimar o LDL-C. Na verdade, a quantificação da Apolipoproteína B (ApoB), padrão-ouro para medição do LDL-C plasmático, não é conveniente para uso rotineiro, pois é cara, trabalhosa e pode ser realizada somente em laboratórios especializados.^25^ Além disso, os ensaios químicos diretos carecem de padronização e seu desempenho depende do tipo de método e reagentes, justificando cautela na interpretação e comparação dos resultados.^26^

Uma das advertências do uso da fórmula de Friedewald é que ela subestima significativamente o LDL-C em pacientes com níveis baixos de LDL-C e/ou níveis elevados de TG.^9^ Como tal, pode não ser ideal para pacientes de risco muito alto, nos quais os níveis recomendados de LDL-C são significativamente baixos.^9,11^ A fórmula de Martin-Hopkins mostrou maior correlação com os níveis de LDL-C medidos por ultracentrifugação, especialmente em valores mais baixos (< 40 mg/dL), conforme demonstrado por Martin et al. em uma análise do ensaio FOURIER.^27,28^ Em pacientes de risco muito alto, descobrimos que a aplicação de Martin-Hopkins mais recente reclassificaria cerca de 20% dos pacientes, anteriormente considerados controlados, no grupo não controlado, enquanto menos de 2% seriam recategorizados como tendo um LDL-C dentro da meta. Sendo assim, estimar rotineiramente o LDL-C pelo método de Martin-Hopkins em pacientes com risco muito alto aumentaria o número de pacientes não controlados, nos quais a intensificação da terapia é justificada. Esses achados estão alinhados com as diretrizes americanas de 2018 que recomendam a medição direta ou o uso da fórmula de Martin-Hopkins para obter níveis de LDL-C quando LDL-C < 70 mg/dL.^11^

Não obstante, deve-se notar que, sob certas circunstâncias, incluindo níveis elevados de TG, diabetes, obesidade e LDL-C muito baixo, tanto o LDL-C calculado como o medido diretamente podem subestimar o risco cardiovascular.^4,29^ Nesses casos, a análise de ApoB, que de outra forma está altamente correlacionada com LDL-C e colesterol não-HDL, é recomendada para avaliação de risco (recomendação classe IC).^4^ As lipoproteínas contendo ApoB desempenham um papel central na iniciação e progressão do processo aterosclerótico.^29^ Embora normalmente 90% das lipoproteínas ApoB circulantes sejam partículas de LDL, nessas circunstâncias mencionadas, o VLDL pode representar uma proporção maior.^30^ Tem havido um interesse crescente na medição direta da ApoB, pois é precisa, barata e não requer jejum.^4,29^ Na verdade, estudos anteriores demonstraram que a ApoB é superior ao LDL-C e ao colesterol não-HDL na previsão de eventos cardiovasculares, sendo o parâmetro mais informativo sobre o benefício da terapia com estatinas.^31,32^

### Pontos fortes e limitações

Vários pontos fortes e limitações deste estudo devem ser reconhecidos. Os estudos observacionais de grande escala mencionados anteriormente são heterogêneos, pois recrutaram pacientes de centros de cuidados primários e secundários ao longo de vários anos, e alguns deles foram produzidos antes da publicação das diretrizes da ESC/EAS de 2019. Como tal, os padrões de prescrição desses estudos e o atingimento das metas de LDL-C podem não refletir completamente as atuais recomendações internacionais. Ao contrário destes estudos, incluímos apenas pacientes com DCVA estabelecida durante um período limitado de tempo em 2022. Além disso, também coletamos dados sobre mudanças terapêuticas, o que fornece informações adicionais sobre o padrão de prescrição de terapia hipolipemiante e adesão às recomendações das diretrizes. Por outro lado, este foi um estudo monocêntrico, com um tamanho amostral pequeno. Na maioria dos pacientes, o infarto agudo do miocárdio foi a primeira manifestação da DCVA. Portanto, o LDL-C basal antes da medicação não estava disponível para a grande maioria, e não foi possível avaliar se os pacientes considerados controlados também tiveram uma redução de LDL-C de pelo menos 50%. Além disso, a intolerância às estatinas foi atribuída de acordo com os registros médicos e pode não corresponder à definição comumente aceita. Finalmente, devido ao desenho retrospectivo do estudo, não temos dados sobre adesão à terapia hipolipemiante ou estilo de vida saudável.

## Conclusão

Neste estudo transversal, menos de um terço dos pacientes pós-infarto do miocárdio, acompanhados na clínica de cardiologia de um hospital terciário, apresentaram valores de LDL-C dentro da meta, com um padrão de prescrição sugerindo uma grande subutilização de terapias prontamente disponíveis. A aplicação da fórmula de Martin-Hopkins para calcular o LDL-C reclassificaria cerca de um quinto dos pacientes presumivelmente controlados no grupo não controlado.

## *Material suplementar

Para informação adicional, por favor, clique aqui.
