# Supplementary material for: Atingimento das Metas de Colesterol LDL em Pacientes com Histórico de Infarto Agudo do Miocárdio: Estudo Transversal do Mundo Real
Source: Arq Bras Cardiol. 2024 Feb 22;121(1):e20230242. [Article in Portuguese] doi: 10.36660/abc.20230242 (PMC11081093; doi:10.36660/abc.20230242)
Supplement: Translation

# Translation: Attainment of LDL-Cholesterol Goals in Patients with Previous Myocardial Infarction: A Real-World Cross-Sectional Analysis

**Authors:** Daniel A. Gomes, Mariana Sousa Paiva, Pedro Freitas, Francisco Albuquerque, Maria Rita Lima, Rita Reis Santos, João Presume, Marisa Trabulo, Carlos Aguiar, Jorge Ferreira, António M. Ferreira, Miguel Mendes

**Affiliations:** 1 Lisboa Portugal Hospital de Santa Cruz – Centro Hospitalar de Lisboa Ocidental, Lisboa – Portugal; 2 Lisboa Portugal Hospital da Luz, Lisboa – Portugal

**Keywords:** Cholesterol, LDL, Atherosclerosis, Secondary Prevention

## Abstract

**Background:**

The European Society of Cardiology guidelines recommend an LDL-cholesterol (LDL-C) < 55 mg/dL for patients with established cardiovascular disease. While the Friedewald equation to estimate LDL-C is still widely used, the newer Martin-Hopkins equation has shown greater accuracy.

**Objectives:**

We aimed to assess: A) the proportion of patients reaching LDL-C goal and the therapies used in a tertiary center; B) the impact of using the Martin-Hopkins method instead of Friedewald’s on the proportion of controlled patients.

**Methods:**

A single-center cross-sectional study including consecutive post-myocardial infarction patients followed by 20 cardiologists in a tertiary hospital. Data was collected retrospectively from clinical appointments that took place after April 2022. For each patient, the LDL-C levels and attainment of goals were estimated from an ambulatory lipid profile using both Friedewald and Martin-Hopkins equations. A two-tailed p-value of < 0.05 was considered statistically significant for all tests.

**Results:**

Overall, 400 patients were included (aged 67 ± 13 years, 77% male). Using Friedewald’s equation, the median LDL-C under therapy was 64 (50-81) mg/dL, and 31% had LDL-C within goals. High-intensity statins were used in 64% of patients, 37% were on ezetimibe, and 0.5% were under PCSK9 inhibitors. Combination therapy of high-intensity statin + ezetimibe was used in 102 patients (26%). Applying the Martin-Hopkins method would reclassify a total of 31 patients (7.8%). Among those deemed controlled by Friedewald’s equation, 27 (21.6%) would have a Martin-Hopkins’ LDL-C above goals.

**Conclusions:**

Less than one-third of post-myocardial infarction patients had LDL-C within the goal. Applying the Martin-Hopkins equation would reclassify one-fifth of presumably controlled patients into the non-controlled group.

**Figure:** A: Density plot of LDL-C estimated by both Friedewald and Martin-Hopkins equations. Overall, 31% and 26% of patients had their LDL-C within the goal, respectively. B: Doughnut chart displaying the prescription of lipid-lowering therapies. Less than two-thirds of patients (64%) were prescribed high-intensity statins. Statin intolerance was reported in five cases.

## Introduction

Low-density lipoprotein cholesterol (LDL-C) is a well-established and modifiable risk factor for atherosclerotic cardiovascular disease (ASCVD).^1-3^ Currently, the 2019 European Society of Cardiology (ESC)/European Atherosclerosis Society (EAS) guidelines for the management of dyslipidemias recommend a reduction of ≥ 50% from the baseline and a goal of LDL-C < 55 mg/dL for secondary prevention (class I-A recommendation).^4^ Non-HDL-cholesterol (non-HDL-C) is regarded as a secondary goal, with a treatment goal of < 85 mg/dL in patients at very high risk of cardiovascular events.^4^ Intensive lipid-lowering therapy is, therefore, the key to reducing the risk of future cardiovascular events.^4^ Previous trials have provided evidence that high-intensity lipid-lowering therapies are safe and more effective than low-intensity drugs at reducing all-cause mortality and recurrent cardiovascular events in patients with ASCVD.^5^ International recommendations advocate the use of high-intensity statins (a surrogate for achieving a reduction of ≥ 50% of LDL-C) as first-line pharmacotherapy to lower LDL-C and cardiovascular risk.^4^ In patients at very high risk of cardiovascular events, including those with established ASCVD, not achieving their set goals on a maximum tolerated dose of a statin, the association of ezetimibe and, if necessary, of a PCSK9 inhibitor is recommended.^4^ Despite being increasingly acknowledged that the largest absolute benefits of lipid-lowering therapies occur in individuals at the greatest risk, such as those with previous ASCVD events, there is yet a significant contrast between the guidelines’ recommendations and real-world clinical practice.^6,7^

Although plasma LDL-C can be directly measured, in clinical practice, it is most often calculated from a standard lipid profile, provided that total cholesterol (TC) is primarily distributed among LDL-C, high-density lipoprotein (HDL-C), and very-low-density lipoprotein (VLDL-C).^4^ Friedewald equation is the most widely used method for estimating LDL-C.^8^ Using this method, LDL-C is calculated by subtracting both HDL-C and triglycerides (TG) /5 (as an estimation for VLDL-C) from TC.^8^ Although convenient, this equation has several limitations. Since a fixed factor of 5 is used to estimate VLDL-C, the Friedewald equation is prone to greater inaccuracy in patients with low LDL-C and/or high TG levels, resulting in a significant underestimation of LDL-C.^9^ Furthermore, the Friedewald equation must be used with caution in patients at high or very high risk of ASCVD due to its significant underestimation of LDL-C, as this prevents physicians from intensifying guideline-recommended lipid-lowering therapy. The Martin-Hopkins equation is a newer method for estimating LDL-C and has shown greater accuracy than Friedewald’s, especially at low LDL-C and high TG.^10^ Unlike the Friedewald equation, Martin-Hopkins divides TG by an adjustable factor based on the patient’s non-HDL-C and TG levels. Although the Friedewald equation is still the most often used, the 2018 American Heart Association/ American College of Cardiology Cholesterol guideline provided a class IIa recommendation (Level of Evidence C) for using either the Martin-Hopkins equation or direct measurement in patients with LDL-C < 70 mg/dL.^11^ Notably, both equations were only validated for patients with TG < 400 mg/dL.^8,10,12^ At higher TG levels, chylomicrons accumulate and may alter the relationship between TG and VLDL-C.^11^ In these circumstances, direct LDL-C measurement should be employed.

## Objectives

The aims of this study were: A) to assess the proportion of patients reaching their LDL-C goal and the therapies used in a tertiary hospital, and B) to assess the impact of using the Martin-Hopkins method instead of the Friedewald equation on the proportion of controlled patients.

## Methods

### Study population

This was a single-center cross-sectional study including consecutive post-myocardial infarction patients followed by 20 different cardiologists (each contributing 20 patients) in a tertiary hospital who had had a clinical appointment after April 2022. Patients were considered eligible if they fulfilled all of the following criteria: A) type 1 acute myocardial infarction ≥ 6 months prior to the appointment; B) available ambulatory fasting lipid profile performed at the hospital’s lab; C) stable lipid-lowering therapy for ≥ 6 weeks prior to the blood analysis; D) measured fasting TG level < 400 mg/dL.

### Demographic, clinical, and laboratory data

Demographic, clinical, and laboratory data, as well as medication, were collected retrospectively from patients’ electronic medical records of clinical appointments. Data regarding therapeutic changes and follow-up scheduling were also collected. Cardiovascular risk factors and myocardial infarction were defined according to current recommendations.^13,14^ Laboratory measurements of TC, HDL-C, and TG were performed from patients’ fasting blood samples at the hospital’s central lab.

This study was conducted according to the amended Declaration of Helsinki. Clinical data was collected by the patient’s attending cardiologist, and irreversibly anonymized prior to its introduction in the database available to the investigators. The patients’ informed consent was waived in the setting of the Quality Certification Program of the National Health Authority for the development of an Internal Audit. There were no missing patients or data. Data was reported according to the RECORD reporting guidelines.^15^

### Lipid-lowering therapies and LDL-C goals

Low-intensity statin was defined as a daily dose of simvastatin 10 mg, pravastatin 10-20 mg, lovastatin 20 mg, or fluvastatin 20-40 mg. Moderate-intensity statin was defined as a daily dose of simvastatin 20-40 mg, atorvastatin 10-20 mg, rosuvastatin 5-10 mg, pravastatin 40-80 mg, pitavastatin 1-4 mg, lovastatin 40 mg, or fluvastatin 80 mg. High-intensity statin was defined as a daily dose of rosuvastatin 20-40 mg or atorvastatin 40-80 mg.^11^ Patients were considered to be intolerant to statins when it was clearly stated in the clinical records.

For each patient, LDL-C levels and attainment of goals were estimated using both Friedewald and Martin-Hopkins equations. According to ESC guidelines, patients were deemed controlled if they had a fasting LDL-C level within the recommended goal (< 55 mg/dL),^4^ while a non-HDL-C level of < 85 mg/dL was a secondary goal.

### Statistical analyses

Categorical variables were reported as numbers and percentages. Continuous variables were described as means and standard deviations for normally distributed variables and medians and interquartile ranges for non-normally distributed variables. The normality of the data was assessed using the Kolmogorov–Smirnov test. Clinical characteristics of the subgroups of interest were compared using the χ2-test and Fisher’s exact test (when applicable) for dichotomous variables, and the unpaired student’s t-test or Mann-Whitney U test was used (when applicable) for continuous variables. Univariable and multivariable logistic regression models were used to explore factors associated with no prescription of high-intensity statins and no attainment of LDL-C goals. Only variables with a p-value < 0.05 were included in the multivariable model. A two-tailed p-value of < 0.05 was considered statistically significant for all tests. All analyses were performed using the IBM® SPSS® Statistics software (version 26.0).

## Results

### Patients’ characteristics and medication

A total of 400 post-myocardial infarction patients were included in the study. The population was comprised of 20 groups of 20 consecutive patients, each cohort being followed up by a different cardiologist. Overall, the last myocardial infarction had occurred a median of five years (IQR 2-12) before the patient’s appointment, and the median clinical practice experience of the attending physicians (i.e., years from medical school graduation) at that time was seven years (IQR 5-26). Patients’ demographic and clinical characteristics are detailed in Table 1 and Supplementary Table 1.

**Table 1:** Demographic, clinical, and laboratory characteristics of the population under study

		All (N = 400)	No high-intensity statin therapy (N = 144)	High-intensity statin therapy (N = 256)	p-value
**Age, years**		67±13	72±12	65±13	< 0.001
**Male sex**		307 (76.8%)	113 (78.5%)	194 (75.8%)	0.541
**Arterial hypertension**		266 (66.5%)	100 (69.4%)	166 (64.8%)	0.349
**Type 2 diabetes**		125 (31.3%)	50 (34.7%)	75 (29.3%)	0.261
**Smoking history**		228 (57.0%)	74 (51.4%)	154 (60.1%)	0.089
	Active smoker	70 (17.5%)	20 (13.9%)	50 (19.5%)	0.154
**Chronic kidney disease** *		72 (18.0%)	32 (22.2%)	40 (15.6%)	0.099
**Years since last MI**		5 (2-12)	10 (5-16)	3 (1-9)	< 0.001
**ASCVD in other territories**					
	Previous stroke	18 (4.5%)	8 (5.6%)	10 (3.9%)	0.445
	Peripheral artery disease	49 (12.3%)	18 (12.5%)	31 (12.1%)	0.909
**Recurrent CV events**		93 (23.3%)	38 (26.4%)	55 (21.5%)	0.265
**Serum lipid profile**					
	Total cholesterol, mg/dL	135 (118-155)	145 (129-166)	130 (114-148)	< 0.001
	HDL-C, mg/dL	45 (38-53)	46 (38-55)	44 (37-52)	0.106
	LDL-C, mg/dL (Friedewald equation)	64 (50-81)	72 (57-92)	62 (47-75)	< 0.001
	Non-HDL-C, mg/dL	89 (72-110)	97 (79-118)	86 (69-103)	< 0.001
	TG, mg/dL	107 (82-153)	107 (84-157)	107 (80-153)	0.776

Values are presented as mean ± standard deviation, median (interquartile range), or n (%).

* Chronic kidney disease defined as an estimated glomerular filtration rate < 60 mL/min/1.73 m^2^ (CKD-EPI equation). ASCVD: atherosclerotic cardiovascular disease; CV: cardiovascular; HDL-C: high-density lipoprotein cholesterol; LDL-C: low-density lipoprotein cholesterol; MI: myocardial infarction; TG: triglycerides.

Using the Friedewald equation, the median estimated LDL-C under therapy was 64 mg/dL (IQR 50-81), whereas it was 69 mg/dL (IQR 54-86) when calculated by the Martin-Hopkins equation. Depending on the estimation method used (Martin-Hopkins or Friedewald), 102 or 125 patients (26% or 31%, respectively) had an LDL-C within the goals [i.e., < 55 mg/dL] (Central Illustration). In total, 88% (n = 110) of patients deemed controlled by the Friedewald equation and all of those with LDL-C within goals as estimated by the Martin-Hopkins equation achieved the secondary non-HDL-C goal.

High-intensity statins were used in 64% of patients, and 26% were prescribed high-intensity statins in association with ezetimibe (Table 2 and Central Illustration). Statin intolerance was reported in 3.5% (n = 5) of patients not taking high-intensity dosages. The group of patients on high-intensity statins (with or without ezetimibe) attained lower plasmatic levels of LDL-C calculated by the Friedewald equation (62 [IQR 47-75] vs. 72 [57-92] mg/dL, p < 0.001), and non-HDL-C (86 [IQR 69-103] vs. 97 [IQR 79-118] mg/dL, p < 0.001) (Table 1). When considering patients under high-intensity statin plus ezetimibe (n = 102), 35% (n = 36) had their LDL-C within the goal, while 11% (n = 11) remained above 100 mg/dL.

**Table 2:** Lipid-lowering therapies

Lipid-lowering therapy		All Cohort (N=400)	LDL-C within goals (N=125) *	LDL-C outside goals (N=275) *	p-value
**Statin monotherapy**		252 (63.0%)	73 (58.4%)	179 (65.1%)	0.199
	Low-intensity	3 (0.8%)	0 (0.0%)	3 (1.1%)	0.241
	Moderate-intensity	95 (23.8%)	20 (16.0%)	75 (27.3%)	0.014
	High-intensity	154 (38.5%)	56 (44.8%)	98 (35.6%)	0.081
**Ezetimibe monotherapy**		3 (0.8%)	1 (0.8%)	2 (0.7%)	0.938
**Statin + ezetimibe**		142 (35.5%)	48 (38.4%)	94 (34.2%)	0.414
	Low-intensity	0 (0.0%)	0 (0.0%)	0 (0.0%)	-
	Moderate-intensity	40 (10.0%)	12 (9.6%)	26 (10.2%)	0.857
	High-intensity	102 (25.5%)	36 (28.8%)	66 (24.0%)	0.773
**Fibrates**		16 (4.0%)	3 (2.4%)	13 (4.7%)	0.271
**PCSK9 inhibitors**		2 (0.5%)	2 (1.6%)	0 (0.0%)	0.035
**No lipid-lowering therapy**		3 (0.8%)	0 (0.0%)	3 (1.1%)	0.241

* LDL-C estimated by the Friedewald equation.

On the multivariable logistic regression model, older patients’ age and attending cardiologist’s years of practice were predictors of not prescribing high-intensity statins (Table 3). Factors associated with the attainment of the LDL-C goals are depicted in Supplementary Table 2.

**Table 3:** Univariable and multivariable analyses exploring factors associated with no high-intensity statin prescription

	Univariable Analysis			Multivariable Analysis		
	OR	95% CI	p-value	Adjusted OR	95% CI	p-value
Age, per year	1.045	1.027-1.064	< 0.001	1.041	1.021–1.060	< 0.001
Male sex	0.858	0.526-1.401	0.541			
Hypertension	0.812	0.524-1.258	0.350			
Type 2 diabetes mellitus	0.779	0.504-1.205	0.262			
Active smoker	0.665	0.378-1.168	0.156			
Years since last MI	0.999	0.996-1.001	0.382			
Chronic kidney disease	0.648	0.386-1.088	0.101			
Peripheral artery disease	0.964	0.519-1.792	0.909			
Cerebrovascular disease	0.691	0.266-1.792	0.447			
Recurrent CV events	0.763	0.474-1.229	0.266			
Attending cardiologists’ clinical experience: > 10 years vs. ≤ 10 years	1.937	1.279-2.932	0.002	1.885	1.230–2.890	0.004

CI: confidence interval; CV: cardiovascular; MI: myocardial infarction; OR: odds ratio.

### LDL-C estimation method

By applying the Martin-Hopkins equation instead of Friedewald’s, a total of 31 patients (7.8%) would be reclassified regarding the attainment of the LDL-C goals. While 27 (21.6%) patients deemed controlled by the Friedewald equation would have a Martin-Hopkins calculated LDL-C above 55 mg/dL, 4 (1.5%) patients previously classified as uncontrolled would have a recalculated LDL-C within goals. (Figure 1) Reclassified patients had higher values of TG (183 [IQR 127-287] vs. 104 [IQR 79-148] mg/dL, p < 0.001) and lower levels of LDL-C as estimated by the Friedewald equation (51 [IQR 46-54] vs. 67 [IQR 61-82] mg/dL, p < 0.001).

**Figure 1:** Sankey diagram showing reclassification of patients according to the attainment of LDL-C goals when applying the Martin-Hopkins equation instead of Friedewald’s. Overall, 22% of the patients who were considered controlled by the Friedewald equation would have a Martin-Hopkins LDL-C above goals, whereas 2% would be recategorized as having LDL-C within goals.

### Medication changes

Adjustment of lipid-lowering therapies was made in 70 (25.5%) of the 275 patients with LDL-C levels above goals. The most frequent medication changes were titration of statin intensity or dose (36, 13.1%) and/or association of ezetimibe (48, 17.5%). Seven patients (2.5%) were referred for PCSK9 inhibitor therapy. The next follow-up appointment was scheduled within a median of 8 months (IQR 6-11).

## Discussion

In this real-world cross-sectional analysis, we report the prescription pattern of lipid-lowering therapies and attainment of LDL-C goals in patients with prior myocardial infarction followed in a Cardiology clinic of a tertiary Hospital. The main results can be summarized as follows: (1) There was a significant underutilization of readily available lipid-lowering therapies, with high-intensity statins being prescribed in less than two-thirds of the patients; (2) Only one in every three patients reached their LDL-C goal; (3) Applying the Martin-Hopkins equation instead of Friedewald’s would reclassify more than 20% of presumably controlled patients into the non-controlled group.

### Prescription pattern and attainment of LDL-C goals

LDL-C is a major determinant of cardiovascular risk and has long been a primary treatment goal in clinical recommendations.^4^ In fact, every 38.7 mg/dL absolute reduction in LDL-C achieved with statins decreases major vascular events by 22% and all-cause mortality drops by 10%.^16^ Current guidelines advocate the use of lipid-lowering therapy (namely high-intensity statins), if not contraindicated, in all patients with established ASCVD irrespectively of baseline LDL-C level to reduce morbidity and mortality, aiming for a goal of LDL-C < 55 mg/dL and a reduction of at least 50%.^4^ It is important to acknowledge that these goals are difficult to obtain with statin monotherapy and combination therapy with ezetimibe is often necessary.^4^ Should LDL-C levels remain above goals, association with a PCSK9 inhibitor is recommended.^4^

In this contemporary study of patients in ASCVD secondary prevention, 64% were prescribed high-intensity statins, and only about 31% had a controlled LDL-C, even if statin intolerance was reported in just over 1%. Furthermore, despite having found that most patients did not have LDL-C within the goal, no more than one-fourth was under high-intensity statin plus ezetimibe, and only two out of 36 (6%) eligible patients, according to the ESC/EAS guidelines, were taking a PCSK9 inhibitor. These results outline the large gap between societal guidelines and real-world clinical practice and, although discouraging, are in line with other previous observational studies: (i) EUROASPIRE V survey reported that only 29% of patients with established coronary heart disease followed in European centers had an LDL-C < 70 mg/dL (previously recommended by the 2016 ESC/EAS guidelines);^17^ (ii) DA VINCI study, including patients in primary and secondary prevention, showed that one-third of them reached the 2019 ESC/EAS LDL-C recommended goals;^18^ (iii) in the baseline analysis of the SANTORINI study, only 21% of patients with established ASCVD followed in primary or secondary care sites across Europe had LDL-C < 55 mg/dL;^19^ (iv) in the LATINO study, including patients followed in Portuguese primary and secondary care sites over 20 years, only 10% of very-high-risk patients achieved the 2016 ESC/EAS LDL-C goal.^20^ Contrarily to what is advocated in the guidelines, high-intensity statins were also significantly underutilized in these large-scale studies, with their use ranging from 12% in LATINO to 50% in the EUROASPIRE V survey in patients at a very high risk of cardiovascular events.^17,20^

Remarkably, older patients, as well as those who had been followed for a longer time, were less likely to receive higher-intensity therapies. At the same time, as opposed to EUROASPIRE V, no sex-related differences in prescription patterns or attainment of LDL-C goals were registered. Furthermore, the attending cardiologist’s years of practice were one of the predictors of not prescribing high-intensity statins. It is possible that more-experienced physicians were less likely to adhere to newer societal guidelines and more likely to rely on clinical evidence that is not up to date, as hypothesized previously.^21,22^

Only roughly 25% of uncontrolled patients had their lipid-lowering medication up-titrated, and the next follow-up appointment was scheduled within a median time of 8 months, despite guidelines recommending reassessing LDL-C within 4-6 weeks and, if necessary, intensifying therapy.^4^ These findings highlight the concept of therapeutic inertia, which is defined as "failure to advance or intensify therapy when therapeutic goals are not reached."^23^ The drivers of therapeutic inertia can be divided into three categories: provider-related (time constraints, competing demands, and lack of knowledge), patient-related (multimorbidity, concerns over side effects, misunderstanding of treatment regimens), and system-related (healthcare issues and costs).^23,24^ Therapeutic inertia may increase the risk of preventable disease-related complications, and therefore, all efforts should be employed to reduce it.^24^

### LDL-C estimation method

The 2019 ESC/EAS guidelines offer no direction on the most suitable method of evaluating LDL-C (direct measurement vs. calculated).^4^ Its accurate assessment is, however, essential as treatment decisions are often based on the achievement of a specific goal. Despite direct methods being increasingly available, the 1972 Friedewald equation is still the most widely used to estimate LDL-C. In fact, Apolipoprotein B (ApoB) quantification, the gold standard for plasma LDL-C measurement, is not convenient for routine use as it is expensive, laborious, and can only be performed in specialized laboratories.^25^ Furthermore, direct chemical assays lack standardization, and their performance depends on the type of method and reagents, warranting caution when interpreting and comparing the results.^26^

One of the caveats of using the Friedewald equation is that it significantly underestimates LDL-C in patients with low LDL-C and/or high TG levels.^9^ As such, it may not be ideal for very-high-risk patients in whom the recommended LDL-C levels are significantly low.^9,11^ The Martin-Hopkins equation has shown a greater correlation with LDL-C measured levels by ultracentrifugation, especially at lower values (< 40 mg/dL), as demonstrated by Martin et al. in an analysis of the FOURIER trial.^27,28^ In very-high-risk patients, we found that applying the newer Martin-Hopkins would reclassify roughly 20% of previously deemed controlled patients into the non-controlled group, while less than 2% would be recategorized as having an LDL-C within goals. Accordingly, routinely estimating LDL-C by the Martin-Hopkins method in patients at very high risk would increase the number of uncontrolled patients with whom therapy intensification is warranted. These findings are in line with the 2018 American guidelines that recommend either direct measurement or the use of the Martin-Hopkins equation to obtain LDL-C levels when LDL-C < 70 mg/dL.^11^

Notwithstanding, it should be noted that under certain circumstances, including elevated TG levels, diabetes, obesity, and very low LDL-C, both calculated and directly measured LDL-C may underestimate the cardiovascular risk.^4,29^ In such cases, ApoB analysis, which is otherwise very highly correlated to LDL-C and non-HDL-C, is recommended for risk assessment (class IC recommendation).^4^ ApoB-containing lipoproteins play a leading role in the initiation and progression of the atherosclerotic process.^29^ Although typically 90% of the circulating ApoB lipoproteins are LDL particles, under those mentioned circumstances, VLDL may represent a greater proportion.^30^ There has been an increasing interest in the direct measurement of ApoB as it is accurate, inexpensive, and does not require fasting.^4,29^ In fact, previous studies have shown that ApoB is superior to LDL-C and non-HDL-C for predicting cardiovascular events and is the most informative parameter on the benefit of statin therapy.^31,32^

### Strengths and limitations

Several strengths and limitations of this study must be acknowledged. The large-scale observational studies mentioned previously are heterogeneous as they recruited patients from both primary and secondary care sites over several years, and some of them were produced before the publication of the 2019 ESC/EAS guidelines. As such, these studies’ prescription patterns and attainment of LDL-C goals may not completely reflect current international recommendations. Unlike these studies, we only included patients with established ASCVD during a limited period of time in 2022. Furthermore, we also collected data regarding therapeutic changes, which provides additional insights into the pattern of lipid-lowering therapy prescription and adherence to guidelines’ recommendations. On the other hand, this was a single-center study with a small sample size. In most patients, acute myocardial infarction was the first manifestation of ASCVD. Therefore, baseline LDL-C before medication was unavailable for the vast majority, and it was not possible to assess whether patients deemed controlled also had a reduction of LDL-C of at least 50%. Also, statin intolerance was ascribed according to physician records and may not correspond to the commonly accepted definition. Finally, due to the study’s retrospective design, we have no data regarding adherence to lipid-lowering therapy or a healthy lifestyle.

## Conclusion

In this cross-sectional study, less than one-third of post-myocardial infarction patients followed in a tertiary hospital’s Cardiology clinic had LDL-C values within the goal, with a prescription pattern suggesting a large underutilization of readily available therapies. Applying the Martin-Hopkins equation to calculate LDL-C would reclassify roughly one-fifth of presumably controlled patients into the non-controlled group.

## *Supplemental Materials

For additional information, please click here.
